# Shear Stress-Dependent Modulation of Endothelin B Receptor: The Role of Endothelial Glycocalyx Heparan Sulfate

**DOI:** 10.3390/cells14141088

**Published:** 2025-07-16

**Authors:** Camden Holm, Son Nam Nguyen, Solomon A. Mensah

**Affiliations:** 1 Biomedical Engineering Department, Worcester Polytechnic Institute, Worcester, MA 01609, USA; cholm@wpi.edu (C.H.); snnguyen@wpi.edu (S.N.N.); 2 Department of Mechanical and Materials Engineering, Worcester Polytechnic Institute, Worcester, MA 01609, USA

**Keywords:** endothelial glycocalyx, mechanotransduction, heparan sulfate, endothelin-1, endothelin B receptor, vascular disease, shear stress

## Abstract

The endothelial glycocalyx (GCX) plays a crucial role in vascular health and integrity and influences many biochemical activities through mechanotransduction, in which heparan sulfate (HS) plays a major role. Endothelin-1 (ET-1) is a potent vasoregulator that binds to the endothelin B receptor (ETB) on endothelial cells (ECs), stimulating vasodilation, and to the endothelin A receptor on smooth muscle cells, stimulating vasoconstriction. While the shear stress (SS) dependence of ET-1 and HS is well documented, there is limited research documenting the SS dependence of the ETB. Understanding the SS dependence of the ETB is crucial for clarifying the role of hemodynamic forces in the endothelin system. We hypothesize that GCX HS regulates the expression of the ETB on the EC surface in an SS-dependent manner. Human lung microvascular ECs were exposed to SS in a parallel-plate flow chamber for 12 h. Damage to the GCX was simulated by treatment with 15 mU/mL heparinase-III during SS exposure. Immunostaining and qPCR were used to evaluate changes in ET-1, ETB, and HS expression. Results indicate that ETB expression is SS sensitive, with at least a 1.3-fold increase in ETB protein expression and a 0.6 to 0.4-fold-change decrease in ETB mRNA expression under SS. This discrepancy suggests post-translational regulation. In some cases, enzymatic degradation of HS attenuated the SS-induced increase in ETB protein, reducing the fold-change difference to 1.1 relative to static controls. This implies that ETB expression may be partially dependent on HS-mediated mechanotransduction, though inconclusively. Furthermore, ET-1 mRNA levels were elevated two-fold under SS without a corresponding rise in ET-1 protein expression or significant impact from HS degradation, implying that post-translational regulation of ET-1 occurs independently of HS.

## 1. Introduction

The vascular endothelial glycocalyx (GCX) consists of a carbohydrate-rich layer and a transmembrane backbone coating the entire surface of endothelial cells (ECs) [1,2]. It is connected to the cytoskeleton and plays a crucial role in vascular permeability and endothelial integrity [3,4,5,6]. The GCX is responsible for EC mechanosensing, where hemodynamic forces are converted into cellular signals, inducing biochemical processes [2,4,5]. Its functions have only recently become the subject of in-depth research. Diseases such as atherosclerosis [7,8] and hypertension [9,10] are known to lead to damage to the GCX, adversely affecting vascular health [3]. The GCX is comprised of a backbone of various transmembrane and membrane-bound proteoglycans and glycoproteins, such as syndecans [11] and glypicans [12], which are attached to glycosaminoglycans (GAGs) by attachment sites on specific extracellular domains [2]. Heparan sulfate (HS) proteoglycans, including syndecan-1 (*SDC1*), perlecan (*HSPG2*), and glypican-1, are the most abundant proteoglycans present in the GCX [1]. Proteoglycan expression is dynamically regulated by external stimuli, and variations in shear stress (SS) magnitude can significantly alter their expression, thereby affecting their functional roles [1,11,13]. Two common HS-containing proteoglycans are *SDC1* and *HSPG2*. Along with glypican-1, these have mechanosensory roles in the GCX, modulating cell behavior and proliferation via translation of mechanical stimulus to the cytoskeleton [2,14]. Studies have shown that the GCX and specifically heparan sulfate proteoglycans (HSPGs) play a role in regulating vascular tone through hemodynamic shear stress by production of nitric oxide (NO) [11,12,15,16].

The primary GAG chains that bind to proteoglycans in the GCX include HS, chondroitin sulfate, hyaluronan, sialic acids, and dermatan sulfate [2]. These proteoglycan and GAG complexes form an intricate network of interconnected carbohydrates creating an interface between the EC surface and blood [1]. HS is the most abundant GAG in the GCX and plays a significant role in mechanotransduction properties of the GCX. HS expression on the EC surface is known to be increased compared with static culture when cells are exposed to laminar SS, simulating the in vivo environment and improving the mechanotransduction response of the ECs [17]. Also, HS recovery after damage is known to be improved by exposure to SS [18]. HS is degraded by heparinase-III (Hep-III), an HS cleaving enzyme, which has been shown to impair the mechanotransduction response in ECs [11]. This is demonstrated by the inhibition of NO production in bovine aortic ECs following treatment with 15 mU/mL Hep-III [11,15].

GCX integrity is negatively impacted by changes to hemodynamic flow. This is known to augment HSPG synthesis in ECs, leading to changes in HSPG mRNA expression and therefore HSPG transcription [13]. The biosynthesis of HSPGs begins with initial synthesis in the rough endoplasmic reticulum, followed by the addition of HS chains and multiple sulfation modifications during transport to the EC surface [13]. Several studies have investigated the SS response of HS and associated proteoglycans, and they suggest that changes in individual HSPG expression vary with dependence on SS magnitude and duration as well as cell type [17,19,20,21]. Sulfation of HS typically occurs by 2-O-sulfotransferase attaching a sulfate group, potentially followed by 3-O-sulfotransferase or 6-O-sulfotransferase modification during transport to the EC surface. The many possible sulfation patterns lead to immense diversity in HS structure and function on the EC surface [13]. Heparinase activity is known to increase under trauma conditions, causing HS remodeling and leading to reduced levels of 3-O-sulfated HS tetramers, which is thought to reduce antithrombin activity [22,23]. Known sulfation modifications to HS occur in disease states, including reduced 3-O-sulfation, which has a high affinity for antithrombin [23]. However, the specific modifications to HSPGs and HS sulfation patterns are very complex, and direct effects from SS on HS biosynthesis are not yet well understood. Other pathologies, such as inflammation and oxidative stress, have been suggested to cause HS and GCX sulfation changes and structural modifications, but the specific modifications and interactions leading to these changes have not yet been clarified [13,24].

Endothelin-1 (ET-1) is a potent vasoconstrictive and vasodilative peptide that plays a significant role in many cardiovascular-related conditions. Elevated ET-1 has been noted in patients with moderate-to-severe hypertension and atherosclerosis, implicating ET-1 in the pathogenesis of these conditions [10]. It is mainly produced by ECs and secreted into the extracellular space to bind to either the endothelin B receptor (ETB) on ECs, stimulating vasodilation, or the endothelial A receptor on smooth muscle cells, stimulating vasoconstriction [25]. Reports indicate that the expression of ET-1 is shear stress (SS) magnitude and time-dependent and that ET-1 expression is NO-mediated [25,26,27]. Extensive studies have investigated the SS dependence of ET-1, largely motivated by apparent conflicting results in early research. Multiple groups have demonstrated that ET-1 release varies across different cell types and is influenced by both the magnitude and duration of SS. These studies suggest that SS-induced modulation of ET-1 is mediated through 3′,5′-cyclic monophosphate and protein kinase C activation, with the upregulation of these signaling pathways likely driven by changes in NO production [25,26,28].

ETB, a plasma-membrane-bound G-protein-coupled receptor (GPCR) on both ECs and smooth muscle cells [29,30], has a high affinity for ET-1 [31,32,33]. It is present on both ECs and smooth muscle cells, and stimulation of ETB leads to a complex cellular response including vasodilation through NO and prostaglandin release as well as G protein activation [30,34]. This response differs depending on the location of ETB, whether found on the EC surface or on smooth muscle cells [34]. Studies have demonstrated the flow sensitivity of GPCRs, though few have investigated the flow sensitivity of ETB [35]. Morawietz et al. demonstrated ETB upregulation in human umbilical vein endothelial cells (HUVECs) in response to long-term (24 h) laminar SS exposure at 1, 15, and 30 dynes/cm^2^. Upregulation was found to occur by an NO- and protein kinase C-dependent mechanism. However, the effects of other SS magnitudes on ETB expression are not well understood [25]. To date, only a few studies have reported SS-dependent regulation of ETB [36,37]. ETB may interact with other EC surface proteins, which include some components of the GCX.

Both ETB and GCX mediate nitric oxide (NO), which is mainly produced by endothelial nitric oxide synthase (eNOS). The GCX mediates NO production by mechanotransduction of stretching and flow-induced forces via proteoglycans, and removal of HS and HSPGs reduces NO production significantly [12,38]. NO is vital for regulation of vascular tone, being activated by the signaling cascade induced by ET-1 binding to ETB and causing vasodilation by decreasing Ca^2+^ entry into smooth muscle cells [30]. The effects of NO on vascular tone regulation by ETB may be affected by HS degradation. Conversely, healthy levels of HS may cause better regulation of vascular tone by ETB. Since the endothelial GCX is known to mediate NO production, it could play a significant role in ETB regulation [38].

Additionally, ET-1 is known to respond to cytoskeletal changes and damage. SS-induced changes in cytoskeletal structures as well as stabilization and disruption of the actin cytoskeleton have been shown to result in changes in ET-1 mRNA expression [39]. Mechanotransduction via HS occurs through proteoglycan connections to the protein components of the cortical cytoskeleton and actin stress fibers [40]. Despite ETB not being known to have a direct cytoskeletal connection, mechanical forces transmitted to the cytoskeleton via HS may influence ETB receptor localization and availability via an indirect pathway. HS may also play a role in regulating ET-1 release via cytoskeletal changes from mechanotransduction, potentially modulating ET-1 and ETB binding and/or synthesis.

Given the mechanosensing properties and spatial proximity of HS, its core proteins, and ETB [41,42], we hypothesize that ETB interacts with HS in a flow-dependent manner to regulate its expression on the surface of ECs. The interaction between ETB, ET-1, and the GCX remains unclear and requires further investigation, which is the primary objective of this project. A deeper understanding of the role the GCX plays in modulating ET-1 and ETB expression may lead to novel diagnostic and therapeutic methods for vascular diseases such as hypertension and atherosclerosis.

To investigate the relationship between ETB and HS, healthy and diseased states were simulated using various SS magnitudes simulating static, low, physiological, and high shear stress magnitudes. Damage to the GCX was also simulated by dosing the media with Hep-III, an HS-cleaving enzyme, which removed the HS component of the GCX to simulate a damaged or diseased GCX. Changes in the protein and mRNA expression of ET-1, ETB, and HS and its core proteins were evaluated by immunocytochemistry (ICC) and qPCR, respectively.

## 2. Materials and Methods

### 2.1. Cell Culture

Human lung microvascular endothelial cells (HLMVECs) were purchased from Cell Applications Inc. (San Diego, CA, USA; Cat# 540-05a) and were cultured in T-75 flasks using Microvascular Endothelial Cell Growth Medium (MEGM) from Cell Applications Inc. at 37 °C and 5% CO_2_ in a water-jacketed incubator. Once 85% confluent, cells were passaged using 0.25% Trypsin-EDTA from Gibco (Grand Island, NY, USA; Cat# 25200072). Cells were used for experiments at passage 6 (P6).

For exposure to fluid shear stress, cells were seeded onto 24 mm × 60 mm glass coverslips. Prior to seeding, coverslips were UV sterilized and coated with 10 mg/mL fibronectin for 30 min. Cells were thawed and seeded directly onto the coverslips at 250,000 cells/coverslip. Cells were allowed to grow for 24 h before exposure to flow.

### 2.2. Heparinase-III Treatment

To simulate damage to HS, cells were dosed with Hep-III purchased from IBEX Pharmaceuticals (Mount Royal, QC, Cannada; Cat# 60-021) at 15 mU/mL for 2 h prior to exposure to SS. Hep-III was also added to the flow system media at 15 mU/mL for the 12 h duration of the flow experiment.

### 2.3. Flow Experiment

Confluent HLMVECs on coverslips were placed inside a custom parallel-plate flow chamber warmed to 37 °C. The flow system was primed with pre-warmed MEGM at 37 °C and then placed inside an incubator for the 12 h for SS exposure. HLMVECs were exposed to shear stress levels of 5, 15, and 25 dynes/cm^2^ for 12 h. Cell culture media was exchanged for static control samples at the start of the SS experiments and were left in the incubator for the duration of the flow experiment.

### 2.4. Fixation

After exposure to SS, cells were rinsed with 1× PBS and were fixed in 2% formaldehyde and 0.1% glutaraldehyde in PBS for 10 min. Samples were then rinsed three times and stored at 4 °C until immunostaining was performed (<1 week).

### 2.5. Immunostaining

Sample coverslips were split into multiple equal sections for immunostaining for ET-1, ETB, and HS. Samples were rinsed with 2% BSA in PBS 3 times for 5 min, then permeabilization of ET-1 and ETB samples was performed in 0.1% Triton X-100 in PBS for 20 min, followed by 3 washes for 5 min each in 2% BSA in PBS. Blocking was performed using 10% goat serum in PBS for one hour. Primary antibodies (Endothelin 1 Monoclonal Antibody, Invitrogen, Carlsbad, CA, USA, Cat# MA3-005; Endothelin B Receptor Polyclonal Antibody, Invitrogen Cat# PA3-066; Ab Heparan Sulfate, purified (clone F58-10E4), AMSBIO, Abingdon, UK, Cat# 370255-1) were prepared in antibody diluent containing 0.1% BSA in PBS and applied to the respective samples at a concentration of 1:500 for ET-1 and HS and 1:1000 for ETB. Negative control samples (shown in Appendix A.2) received only antibody diluent for this step. Samples were incubated overnight at 4 °C in the dark. Next, samples were rinsed 3 times for 5 min each with 2% BSA in PBS, and secondary antibodies (Goat anti-Mouse IgG1 Cross-Adsorbed Secondary Antibody, Invitrogen Cat# A-21121; Goat anti-Rabbit IgG (H + L) Highly Cross-Adsorbed Secondary Antibody, Invitrogen Cat# A-11036; Goat anti-Mouse IgG, IgM (H + L) Secondary Antibody, Invitrogen Cat# A10680) were applied for one hour at room temperature in the dark at concentrations of 1:400 for ET-1 and ETB and 1:5000 for HS secondary antibodies. After rinsing 3 times for 5 min with 2% BSA in PBS and 3 times with deionized water, coverslips were mounted with Fluoromount-G mounting medium with 4′,6-diamidino-2-phenylindole (DAPI) (Invitrogen Cat# 00-4959-52) onto microscope slides and stored at room temperature in the dark overnight to cure the mounting medium. Samples were kept at 4 °C in the dark until imaging.

### 2.6. Imaging

A Nikon Eclipse Ti2 spinning-disk confocal microscopy system (Tokyo, Japan) with a CSU-W1 Confocal Scanner Unit from Yokogawa (Musashino, Tokyo, Japan) was used to capture z-stack images of samples for quantification using an Olympus plan-achromat 60× 1.4 N.A. oil objective. Z-stack images were taken at a randomly selected area on the sample within the fluid flow path with a slice thickness of 0.2 µm. In total, 31 slices were taken for each image by finding the approximate middle of the cell layer and imaging 3 µm in either direction from the center of the sample. Each z-stack was captured as a 16-bit image with a resolution of 2048 × 2048 pixels. Imaging parameters, including laser power and exposure duration, were kept consistent for all independent experiments.

### 2.7. Image Analysis

Fluorescent confocal images were analyzed using a custom image analysis pipeline using ImageJ FIJI (v1.54f) [43] and CellProfiler (v4.2.8) [44]. The sum projections of z-stack images were generated using ImageJ FIJI and saved as uncompressed TIFF files. These sum-projection images were loaded into CellProfiler for cell segmentation and analysis of fluorescent intensity. The CellProfiler pipelines used in this analysis are available on the data repository FigShare at the DOI listed in the Data Availability Statement. Fluorescent intensity was calculated per cell area for ET-1 and ETB samples and field of view (FOV) for HS samples, and a fold-change (FC) calculation was performed to compare the change in integrated intensity between the flow and diseased conditions and the static control for each sample.

Upon visual inspection, confocal images show little difference in fluorescent intensity between samples, and conclusions cannot be made in this manner. Rigorous computational analysis of fluorescent intensity in confocal images must be performed to properly analyze changes in fluorescent intensity in samples due to the various treatments. A version of this analysis has been performed previously by Mensah et al. and Cancel et al. [45,46]. However, for this study, a more robust analysis method was developed to better assess any potential differences in expression via fluorescent intensity and eliminate sources of bias in image analysis. This analysis method utilized ImageJ FIJI [43] to generate sum projections of z-stacks and CellProfiler [44] to segment cells within the field of view (FOV) and measure the integrated intensity (the sum of all pixel intensity values) for each cell to obtain an average intensity value per cell for ET-1 and ETB samples. This same method was utilized for HS samples, but the CellProfiler pipeline was tuned to select all areas of HS expression within the FOV, which is not localized to the cells, to obtain an average intensity value per image. To ensure a large enough sample size, at least 3 images for each ET-1 and ETB sample and at least 6 images for each HS sample were captured at random locations within the flow path and analyzed for each experiment. This method allows for unbiased image analysis by allowing for identical and minimal processing of all images and generates numerical values for each image for proper statistical analysis.

### 2.8. qPCR

qPCR was performed alongside immunostained samples using the SYBR Green Fast Advanced Cells-to-CT Kit from ThermoFisher Scientific (Waltham, MA, USA; Cat# A35379). After exposure to SS, cells outside of the flow path were removed from the coverslip using a cell scraper. Visual inspection under a microscope was used to confirm complete removal of cells. Coverslips were rinsed in PBS and cells were lifted from the coverslip using 0.25% Trypsin-EDTA and pelletized in a centrifuge. Cells were then counted and brought to a concentration of 25,000 cells/5 µL in PBS, as directed by the manufacturer. Lysis was performed following the manufacturer’s specifications using 50 µL of lysis solution, 0.5 µL of DNase I, and 5 µL of cell suspension (25,000 cells), followed by gentle mixing and incubation at room temperature for 5 min. A stop solution was added to halt lysis, and the lysate was stored at −20 °C until use (<2 weeks). Reverse transcription (RT) was performed using Fast Advanced RT buffer, enzyme mix, UltraPure™ DNase/RNase-Free Distilled Water from Invitrogen ( Cat# 10977015) and lysate. Controls used were nuclease-free H_2_O, a no-template control, and negative RT control (-RT) (no reverse transcription). The RT reaction was performed in a Bio-Rad DNAEngine Peltier Thermal Cycler (Bio-Rad Laboratories; Hercules, CA, USA) with a Bio-Rad Alpha Unit Block Assembly (Bio-Rad, Cat# ALD1244) by holding at 37 °C for 30 min and was inactivated at 95 °C for 5 min. The resulting cDNA was stored at 4 °C until use (<1 day).

Prior to loading the qPCR plate, master mixes (MMs) were prepared with SYBR Green, primers, and nuclease-free (NF) water. Controls were loaded, followed by 8 µL of MM in each respective well, then 2 µL of cDNA or -RT was loaded into each well. Triplicate samples were used for all samples, and duplicate wells were used for all controls due to a lack of space on the plate. A no-template control (NTC) consisting of only 10 µL of MM and two wells containing NF H_2_O were loaded prior to loading the plate, and two more wells were loaded after the plate was fully loaded to act as additional controls. The qPCR reaction was performed in a Quant Studio 6 Pro Real Time PCR system (Thermo Fisher Scientific, Waltham, MA, USA) protocol, beginning with UDG activation at 50 °C for 2 min, enzyme activation at 95 °C for 10 min, and 40 amplification cycles at 95 °C for 3 s and 60 °C for 30 s. Primers (see Table A1 in Appendix A.1 for primer sequences) for *EDN1*, *EDNRB*, and *HSPG2* were validated prior to use, and the *SDC1* primer used was from Taghavi et al. [47]. All primers were used at a final working concentration of 500 nM. Expression was quantified using the 2^−ΔΔCt^ method [48].

Primers were designed using NCBI Primer-BLAST [49]. Briefly, the gene ID was entered into Primer-BLAST to look for primers common to a group of sequences, then “primer must span an exon-exon junction” was selected. Primer sequences that had short product lengths, spanned an exon–exon junction, had GC content between 40 and 60%, were 18–24 bp in total length, contained balanced AT and GC content, and avoided runs of 4 or more bps were selected. Primers were ordered from Sigma-Aldrich (St. Louis, MO, USA). Primer sequences were validated on HLMVECs following the qPCR protocol above and using a range of primer concentrations from 100 nM to 10 uM along with -RT, NTC, and NF H_2_O controls to determine the best primer concentration and most effective primer. Melt curves were analyzed to confirm that a single sharp peak had appeared and minimal self-annealing had occurred. Primers that consistently had Ct values below 32 were considered acceptable, but most primers produced average Ct values below 25.

### 2.9. Statistics and Data Representation

All data is presented as the group mean, and variance is presented as the standard error of the mean. At least 5 independent experiments were used as numerical replicates for each experimental group. Multiple-comparison analysis via ordinary one-way ANOVA tests was used to determine statistical significance between all sample means in an experiment when comparing control and enzyme-treated samples with SS exposure to a static control. In the case of static control and enzyme treatment comparisons, an unpaired parametric *t*-test was performed to determine statistical significance between the two samples. *p*-values less than 0.05 were considered significant. All data presented graphically was analyzed using GraphPad Prism (Version 10.3.1 for Windows, GraphPad Software, www.graphpad.com).

## 3. Results

To investigate the relationship between ET-1, ETB, and HS, HLMVECs were exposed to uniform flow (UF) of varying SS magnitudes and treated with Hep-III for HS degradation. HLMVECs were selected because of our interest in studying hypertension and our strong understanding of the culture behavior of these cells. Immunostaining and qPCR were used for analysis of changes in ET-1, ETB, and HS expression. Confocal images of immunostained samples were analyzed using a custom pipeline created in ImageJ FIJI and CellProfiler, and analysis of mRNA expression via qPCR was performed using the 2^−ΔΔCt^ method [44,48,50]. Representative negative control images for all immunostained samples are shown in Appendix A.2. The following sections describe the effects of 12 h of SS exposure combined with 15 mU/mL Hep-III (Hep-III) treatment following a 2 h pre-exposure period (14 h of total Hep-III exposure).

### 3.1. ET-1 Results

**Figure 1:**
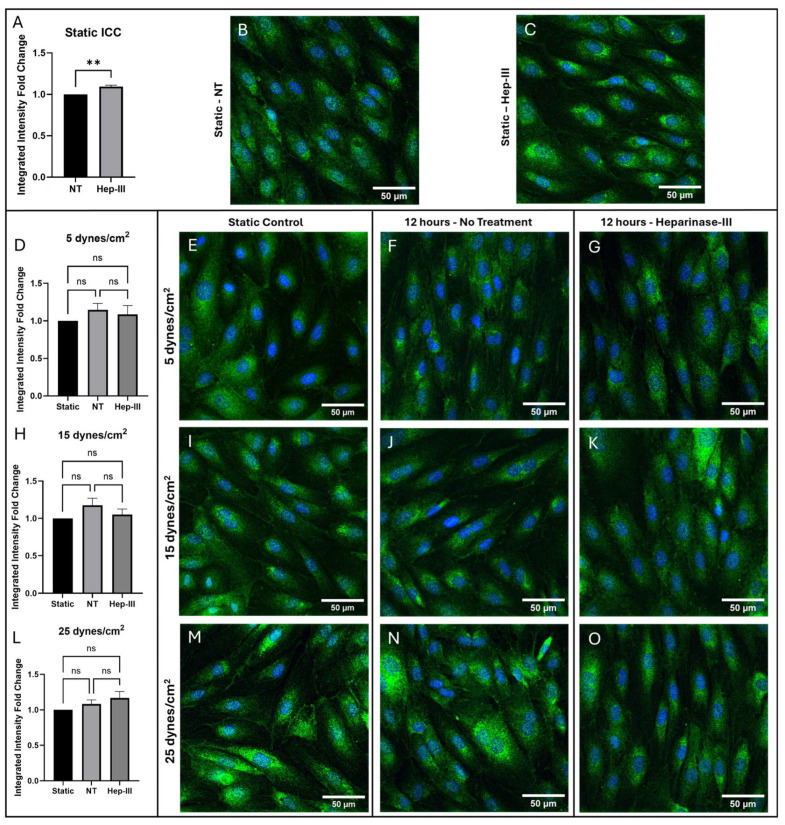
Immunostaining results for ET-1 in cells exposed to 0, 5, 15, and 25 dynes/cm^2^ of uniform SS. Green represents ET-1, while blue represents cell nuclei. (**A**,**D**,**H**,**L**) Integrated intensity fold-change analysis of cells positive for ET-1 in NT and Hep-III conditions compared to the NT static condition. Representative confocal microscopy images of static controls that were compared to all SS magnitudes (**B**,**E**,**I**,**M**), and samples exposed to 5 (**F**,**G**), 15 (**J**,**K**), and 25 (**N**,**O**) dynes/cm^2^ of uniform SS stained for ET-1 under NT and Hep-III conditions. A static Hep-III treated sample is shown in (**C**). Statistical significance indicated by asterisks (** *p* < 0.01); ns, not significant (*p* > 0.05).

**Figure 2:**
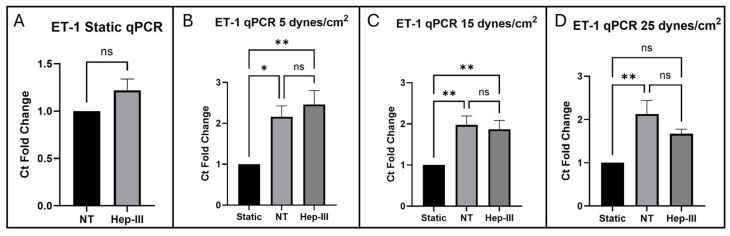
qPCR results for *EDN1* expression in cells exposed to (**A**) 0, (**B**) 5, (**C**) 15, and (**D**) 25 dynes/cm^2^ of uniform SS. Statistical significance indicated by asterisks (* *p* ≤ 0.05; ** *p* < 0.01); ns, not significant (*p* > 0.05).

Figure 1 presents confocal images of immunostained samples alongside the analysis of integrated intensity foldchange and Figure 2 presents the threshold cycle (Ct) fold change from qPCR in cells exposed to 0, 5, 15, and 25 dynes/cm^2^ of uniform SS. The static control images in Figure 1B,E,I,M are representative of the static control images compared to each SS magnitude to generate the graphs in Figure 1A,D,H,L. Following exposure to 15 mU/mL Hep-III for 14 h, ET-1 protein expression showed a slight increase under static conditions (*p* = 0.0037; Figure 1A). *EDN1* gene expression did not significantly increase under enzyme exposure (*p* = 0.0958; Figure 2A).

ET-1 protein expression did not significantly change under low-magnitude SS (Figure 1F,G) as shown by the graph in Figure 1D (*p* = 0.4304). However, ET-1 gene expression was markedly upregulated in both no-treatment (NT) and Hep-III conditions, with fluorescent intensity values more than doubling compared with static culture samples (*p* = 0.0021; Figure 2B). *EDN1* gene expression was unaffected by the Hep-III treatment, indicating potential HS independence.

Under 15 dynes/cm^2^ of uniform SS, HLMVECs showed no change in ET-1 protein expression in both NT (Figure 1J) and HS-degraded (Figure 1K) conditions when compared to static conditions (*p* = 0.2270; Figure 1H). A strong increase in gene expression was observed under SS conditions (*p* = 0.0025; Figure 2C) and, similarly to the lower SS results, this effect was unchanged by degradation of HS, suggesting HS independence in ET-1 gene regulation.

Exposure to 25 dynes/cm^2^ of SS resulted again in no change in ET-1 protein expression under SS conditions (Figure 1N), and this effect was repeated under Hep-III (Figure 1O) treatment (*p* = 0.1665; Figure 1L). At high SS, ET-1 mRNA was again significantly increased (*p* = 0.0030; Figure 2D). This effect was eliminated by HS degradation, indicating a potential role for HS in regulating ET-1 transcription. This increase was similar in magnitude across all SS conditions, strongly demonstrating the SS dependence of ET-1 expression and potentially suggesting HS dependence of ET-1 under high SS magnitudes.

A detailed analysis showed that the integrated intensity fold change of ET-1 protein expression remained unchanged in all SS magnitudes of 5, 15, and 25 dynes/cm^2^ when compared to static control samples. Additionally, application of SS with HS degradation had no significant effect on ET-1 protein expression within the cell at SS levels of 5, 15, or 25 dynes/cm^2^. This result suggests that HS might not be directly involved in the translational or post-translational regulation of ET-1.

Analysis of qPCR results showed that *EDN1* gene expression is significantly upregulated by SS but not significantly affected by Hep-III treatment at 5 and 15 dynes/cm^2^, which is consistent with ICC results. Under uniform SS for 12 h at 5, 15, and 25 dynes/cm^2^, *EDN1* gene expression was significantly increased compared with static, confirming that *EDN1* expression is SS dependent. When exposed to uniform SS at 5 and 15 dynes/cm^2^ in the presence of 15 mU/mL Hep-III, no significant differences were observed compared to the SS control samples. At 25 dynes/cm^2^, *EDN1* expression was decreased in samples dosed with Hep-III compared with untreated samples, with no significance in fold change when compared to static samples. These results taken together could suggest that HS is involved in regulation of ET-1 expression at the transcriptional level when ECs are exposed to high physiological SS and that further investigation is required to understand the mechanisms at play. However, gene upregulation without a corresponding increase in protein expression indicates post-translational regulation, potentially involving processes such as secretion or degradation.

### 3.2. ETB Results

**Figure 3:**
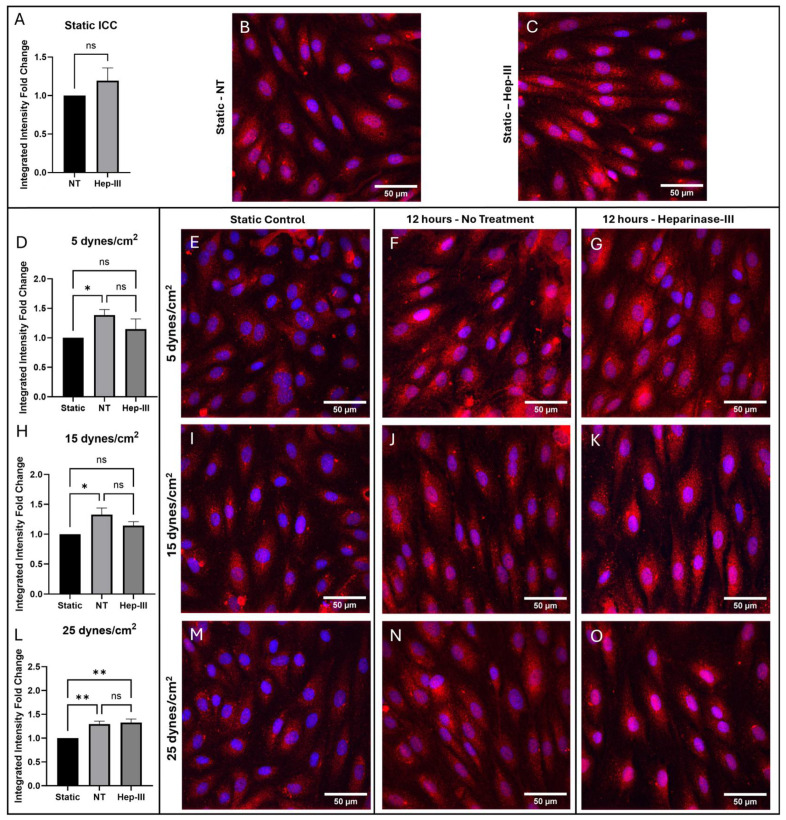
Immunostaining results for ETB in cells exposed to 0, 5, 15, and 25 dynes/cm^2^ of uniform SS. Red represents ETB, while blue represents cell nuclei. (**A**,**D**,**H**,**L**) Integrated intensity fold-change analysis of cells positive for ETB in NT and Hep-III conditions compared to the NT static condition. Representative confocal microscopy images of static controls that were compared to all SS magnitudes (**B**,**E**,**I**,**M**), and samples exposed to 5 (**F**,**G**), 15 (**J**,**K**), and 25 (**N**,**O**) dynes/cm^2^ of uniform SS stained for ETB under NT and Hep-III conditions. A static Hep-III treated sample is shown in (**C**). Statistical significance indicated by asterisks (* *p* ≤ 0.05; ** *p* < 0.01); ns, not significant (*p* > 0.05).

**Figure 4:**
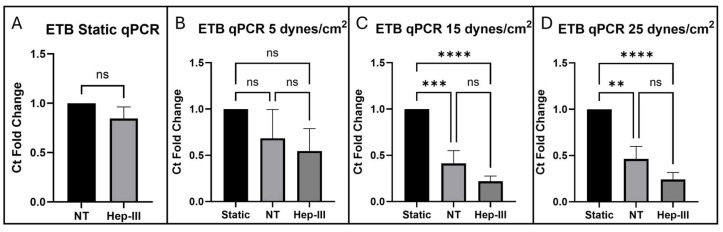
qPCR results for *EDNRB* expression in cells exposed to (**A**) 0, (**B**) 5, (**C**) 15, and (**D**) 25 dynes/cm^2^ of uniform SS. Statistical significance indicated by asterisks (** *p* < 0.01; *** *p* < 0.001; **** *p* < 0.0001); ns, not significant (*p* > 0.05).

Figure 3 describes ETB protein expression under 0, 5, 15, and 25 dynes/cm^2^. The static control images in Figure 3B,E,I,M are representative of the static control images compared to each SS magnitude to generate the graphs in Figure 3A,D,H,L. ETB expression showed no difference in protein expression under enzyme exposure (*p* = 0.3022; Figure 3A) and no change in gene expression under enzyme exposure (*p* = 0.2183; Figure 4A). ETB protein levels increased under SS exposure compared with static samples (Figure 3F) but this increase was abolished by Hep-III (Figure 3G) treatment (*p* = 0.0521; Figure 3D), resulting in no significant difference in ETB expression in Hep-III-treated samples compared to static samples. This demonstrates that ETB is SS dependent in low physiological SS conditions and indicates potential HS dependence for protein regulation. *EDNRB* gene regulation showed a non-significant decrease under low SS conditions, indicating a mild sensitivity to low physiological SS (*p* = 0.3772; Figure 4B).

In Figure 3J, we show a strong increase in ETB protein expression under 15 dynes/cm^2^ of uniform SS compared with static samples (*p* = 0.0255; Figure 3H). This SS-induced increase in ETB was blocked by HS degradation (Figure 3K), possibly indicating HS-dependent expression of ETB. Conversely, *EDNRB* gene expression was drastically decreased under SS exposure (*p* = 0.0001; Figure 4C), and HS degradation did not significantly affect the transcriptional response, indicating potential HS-independent transcription under physiological SS conditions.

ETB expression under 25 dynes/cm^2^ of uniform SS is shown in Figure 3N. Under high physiological SS, ETB protein expression was again observed to increase (*p* = 0.0007; Figure 3L), and Hep-III treatment (Figure 3O) did not affect this increase. Gene expression decreased at high SS and was again not affected by Hep-III treatment, further suggesting HS-independent transcription of ETB (*p* = 0.0001; Figure 4D).

In summary, ETB protein expression was increased significantly in all tested SS conditions. HS-degraded 5 and 15 dyne/cm^2^ samples showed no significant change compared to the static control, showing a reduction in the effect SS on ETB expression. This could indicate that HS is significantly involved in ETB expression and removal of HS affects ETB expression at a post-translational level. However, no statistically significant change in ETB expression was found between NT and Hep-III-treated samples. Additionally, samples exposed to 25 dynes/cm^2^ exhibited significantly increased ETB expression under both control and enzyme-treated conditions. These results indicate that ETB expression is SS dependent. Reduction of the mechanotransduction response by HS degradation may prevent ETB upregulation under low and physiological SS conditions, but this was not a statistically significant result and was not observed under high SS conditions.

*EDNRB* gene expression was shown to be SS dependent and was found to be unaffected by Hep-III exposure under static conditions. Gene expression of *EDNRB* showed a non-significant decrease in fold change compared with the static control when HLMVECs were exposed to low uniform SS, indicating a low sensitivity to mild SS. At 15 dynes/cm^2^, *EDNRB* gene expression was significantly decreased in both control and enzyme-dosed conditions with no significant fold-change difference between static control and enzyme-treated conditions. At high SS (25 dynes/cm^2^), the *EDNRB* gene expression fold change further decreased and was not significantly affected by HS degradation, suggesting that ETB gene expression is SS dependent and HS independent.

### 3.3. HS Results

**Figure 5:**
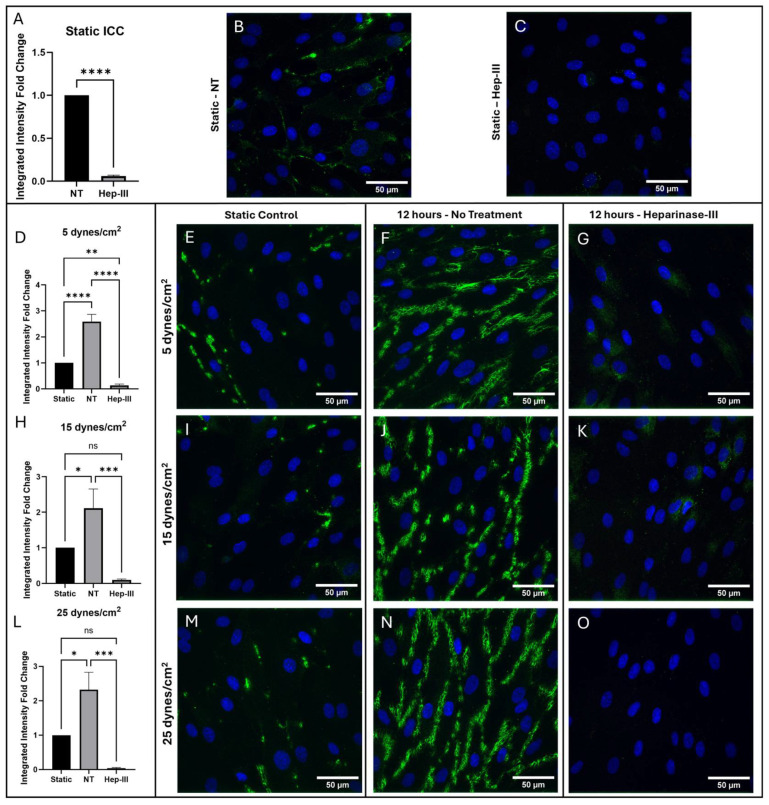
Immunostaining results for HS in cells exposed to 0, 5, 15, and 25 dynes/cm^2^ of uniform SS. Green represents HS, while blue represents cell nuclei. (**A**,**D**,**H**,**L**) Integrated intensity fold-change analysis of cells positive for HS in NT and Hep-III conditions compared to the NT static condition. Representative confocal microscopy images of static controls that were compared to all SS magnitudes (**B**,**E**,**I**,**M**), and samples exposed to 5 (**F**,**G**), 15 (**J**,**K**), and 25 (**N**,**O**) dynes/cm^2^ of uniform SS stained for HS under NT and Hep-III conditions. A static Hep-III treated sample is shown in (**C**). Statistical significance indicated by asterisks (* *p* ≤ 0.05; ** *p* < 0.01; *** *p* < 0.001; **** *p* < 0.0001); ns, not significant (*p* > 0.05).

**Figure 6:**
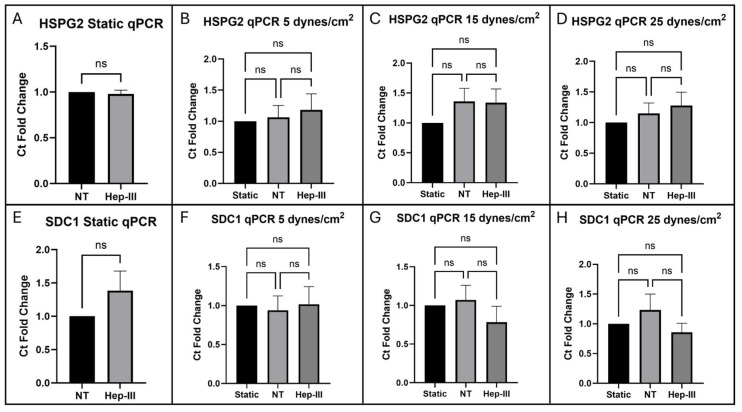
qPCR results for the HSPG perlecan (*HSPG2*) exposed to (**A**) 0, (**B**) 5, (**C**) 15, and (**D**) 25 dynes/cm^2^ of uniform SS and syndecan-1 (*SDC1*) expression in cells exposed to (**E**) 0, (**F**) 5, (**G**) 15, and (**H**) 25 dynes/cm^2^ of uniform SS. ns, not significant (*p* > 0.05).

HS was significantly removed from the cell surface by Hep-III treatment as shown in Figure 5. Confocal images of HS ICC under static culture conditions comparing samples with and without 14 h enzyme treatment are shown (Figure 5B,C), along with a graph depicting the fold change of integrated intensity (Figure 5A). The static control images in Figure 5B,E,I,M are representative of the static control images compared to each SS magnitude to generate the graphs in Figure 5A,D,H,L. Gene expression of *HSPG2* (Figure 6A) and *SDC1* (Figure 6E) was unchanged by enzyme treatment in static conditions.

HS expression on the EC surface significantly increased under 5 dynes/cm^2^ of uniform SS (*p* = 0.0001; Figure 5D). This increase (Figure 5F) was abolished under exposure to Hep-III (Figure 5G), confirming effective degradation of HS by 15 mU/mL Hep-III. No significant changes occurred in *HSPG2* (*p* = 0.7825; Figure 6B) or *SDC1* (*p* = 0.9447; Figure 6F) gene expression under low SS, indicating that mild SS does not lead to HSPG transcription.

Expression of HS on the HLMVEC surface also increased under 15 dynes/cm^2^ (Figure 5J) of uniform SS (*p* = 0.0006; Figure 5H). This effect was again eliminated by the introduction of Hep-III (Figure 5K) to degrade HS. Similarly to low SS exposure, under 15 dynes/cm^2^, no change was observed in *HSPG2* (*p* = 0.3229; Figure 6C) or *SDC1* (*p* = 0.4443; Figure 6G) gene expression, indicating that moderate SS does not lead to increased transcription of HSPGs.

Uniform SS at 25 dynes/cm^2^ also significantly increased HS expression (Figure 5N) on the EC surface, and Hep-III treatment (Figure 5O) completely reduced HS expression (*p* = 0.0002; Figure 5L). Even under high SS, gene expression of the HS core proteins *HSPG2* (*p* = 04882; Figure 6D) and *SDC1* (*p* = 0.3465; Figure 6H) remained unchanged, strongly indicating that HS levels increase due to shear-induced post-transcriptional remodeling.

To summarize, HLMVECs were confirmed to robustly increase expression of HS on the cell surface under all SS conditions as shown by the integrated intensity fold change. This effect was eliminated when HS was degraded with Hep-III. The highest increase in HS expression was observed at 25 dynes/cm^2^, while Hep-III treatment completely eliminated HS. This result demonstrates the effective degradation of HS by Hep-III at 15 mU/mL.

Gene expression fold-change analysis of the HS proteoglycans *HSPG2* and *SDC1* showed no significant effect when HLMVECs were dosed with 15 mU/mL Hep-III for 12 h plus pre-treatment for 2 h. Perlecan showed no fold-change difference between control and enzyme-treated static samples and syndecan-1 showed a slight but non-significant increase in gene expression by fold-change analysis. Gene expression of *HSPG2* was not significantly affected by exposure to SS at 5, 15, or 25 dynes/cm^2^. *SDC1* was also not significantly affected by uniform SS at 5, 15, or 25 dynes/cm^2^, indicating that SS may not induce HS proteoglycan synthesis.

## 4. Discussion

### 4.1. Summary of Results

In this study, we examined how HS and SS influence the expression of ETB on ECs. We hypothesized that SS-driven HS synthesis plays a regulatory role in modulating ETB expression in ECs. We conducted our study using a parallel-plate flow chamber system modeled after a previously developed system to simulate uniform blood flow in vivo [20,51]. HLMVECs were exposed to UF for 12 h and gene and protein expression of ET-1, ETB, and HS were evaluated by comparison to a static control (0 dynes/cm^2^). We chose 5, 15, and 25 dynes/cm^2^ to replicate in vivo conditions for HLMVECs. We chose 5 and 25 dynes/cm^2^ to replicate low and high physiological SS conditions, respectively, as ET-1 release under 5 and 25 dynes/cm^2^ had been characterized and 15 dynes/cm^2^ was chosen to replicate normal healthy physiological conditions [25,26,52].

A summary of the observed effects of combined SS and HS degradation are shown in Table 1 below. Briefly, we observed no change in ET-1 protein expression accompanied by an increase in gene expression under SS conditions, and no difference was observed between NT and Hep-III samples except under 25 dynes/cm^2^ of SS. For ETB, we observed an increase in protein expression and a decrease in gene expression under SS, with some observed effect from HS degradation. Finally, a significant increase in HS on the cell surface was observed under SS, while HS proteoglycan gene expression remained unaffected by SS and Hep-III exposure.

### 4.2. Major Findings and Importance

To confirm that Hep-III treatment did not significantly alter the baseline expression of ET-1, ETB, or HSPGs, comparisons were made between NT and Hep-III-treated samples under static conditions only (Figure 1A, Figure 2A, Figure 3A, Figure 4A and Figure 6A,E). The results of this control comparison show that the introduction of Hep-III to HLMVECs alone is not enough to cause significant protein expression changes in ETB or gene expression changes in *EDN1*, *EDNRB*, perlecan, or syndecan-1. This indicates that changes in protein or gene expression seen under SS conditions can be attributed to combined effects of SS or HS degradation and not by the addition of Hep-III alone.

ET-1 mRNA expression was significantly increased compared with static conditions under SS and was unaffected by HS degradation at 5 and 15 dynes/cm^2^. HS degradation reduced the upregulation of *EDN1* induced by high physiological shear stress (25 dynes/cm^2^). ET-1 protein expression was found to not change across all SS levels despite significant gene upregulation. HS degradation had no significant effect on ET-1 protein expression levels within the cell, which is consistent with gene expression results under 5 and 15 dynes/cm^2^ of uniform SS. This demonstrates that SS upregulates ET-1 transcription through an HS-independent pathway under low and moderate SS. Attenuation of mechanotransduction through HS degradation may disrupt the normal SS-dependent regulation of ET-1 gene expression. This data shows that ET-1 expression is SS dependent, increasing ET-1 mRNA under SS conditions despite no change in protein expression. The lack of a significant change in protein expression accompanying significant gene upregulation suggests post-translational regulation of ET-1 by a mechanism such as degradation or rapid secretion into the surrounding environment.

*EDNRB* gene expression showed a slight, non-significant decrease compared with static control under exposure to 5 dynes/cm^2^, indicating a low sensitivity to mild shear stress. At 15 dynes/cm^2^, *EDNRB* gene expression was significantly decreased, but the transcriptional response was not significantly affected by HS degradation, suggesting HS-independent transcription. At high SS (25 dynes/cm^2^), *EDNRB* gene expression further decreased significantly, and was also not significantly affected by HS degradation, confirming HS-independent transcription of ETB. Protein expression of ETB increased under 5, 15, and 25 dynes/cm^2^ compared with static conditions. Hep-III treatment for HS degradation eliminated the statistical significance of protein upregulation in low and physiological SS conditions but did not affect the change in protein expression under high SS conditions. Based on the data, it appears that ETB expression in response to SS may be dependent on a specific shear stress threshold.

While this result does not confirm that ETB is HS dependent, negation of the SS-induced change in ETB expression by degradation of HS may suggest that the presence of healthy, functional HS may play a role in regulating ETB expression. Overall, ETB expression was found to be SS dependent, and indications of partial HS dependence were observed. *EDNRB* gene expression was found to decrease in a SS-dependent manner independently of HS health, and ETB showed a SS-dependent increase in protein expression. Hep-III treatment, which disrupts HS-mediated mechanotransduction [11], led to a decrease in ETB integrated intensity measured by FC relative to NT conditions, although the change was not statistically significant. However, the statistical significance of the SS-dependent change in ETB expression under healthy HS conditions was eliminated by Hep-III treatment under 5 and 15 dynes/cm^2^ conditions (Figure 3). SS is more responsible for ETB regulation, but this result, shown in Figure 3, implies potential HS dependence. Alternatively, other GAGs maybe be more responsible for ETB modulation by the GCX. Further research is required to elucidate the specific mechanisms involved in this cooperation.

HS expression on the EC surface was significantly increased under all SS conditions (5, 15, and 25 dynes/cm^2^). The highest increase in HS expression occurred under high SS, and Hep-III exposure completely removed the fluorescent signal. Hep-III treatment abolished the increase in HS endothelial surface expression in all treatments, confirming that Hep-III effectively degrades HS at 15 mU/mL. These results suggest that SS exposure increases HS expression post-translationally, likely through increased GCX remodeling via GAG chain length or by reduced turnover [53]. HS gene expression was unchanged by SS exposure, highlighting that HS remodeling, not proteoglycan synthesis, mediates shear-induced changes in EC activity.

### 4.3. Alternative Explanations

The ET-1 regulatory pathway is known to be highly complex, with ET-1 release known to be, among other things, thrombin induced [54], vasopressin induced [55], hypoxia induced [56], and SS dependent [26,27,28]. Under conditions of vascular stress or in the presence of vasoactive substances and inflammatory cytokines such as angiotensin II and tumor necrosis factor-α (TNF-α), ET-1 mRNA is known to be upregulated [30,57]. Under 20 dynes/cm^2^ of SS, ET-1 mRNA is known to increase under less than 2 h of exposure followed by a decrease at exposure durations greater than 2 h [58], further indicating the complexity of the ET-1 regulatory pathway. Under high, uniform physiological SS, ET-1 gene expression was significantly elevated compared with static culture. In contrast, only a non-significant increase was observed after Hep-III treatment. This pattern was not observed at lower SS magnitudes, suggesting that ET-1 regulation under high SS may differ from that under lower SS magnitudes. These findings imply that ET-1 expression in response to high SS involves additional or alternative regulatory mechanisms beyond HS-mediated mechanotransduction alone, pointing toward a more complex, potentially threshold-dependent regulatory pathway for ET-1. Additionally, the SS dependence of ET-1 may be more closely linked to a different mechanotransduction component of the GCX than HS, such as hyaluronic acid (HA) or sialic acids (SAs), which are also known to be involved in mechanotransduction [16].

ETB was found to be SS dependent and may also be partially HS dependent, though our results show no statistically significant difference between NT and Hep-III samples. The opposing trends of decreased gene expression alongside increased protein expression suggest that ETB regulation extends beyond simple HS-mediated SS dependency, potentially involving post-translational mechanisms. The lack of a significant SS-induced change in ETB expression in Hep-III-treated samples, which contrasted with the untreated condition, suggests that HS degradation impaired the mechanotransduction pathway responsible for mediating ETB upregulation under SS. These findings, together with the observed SS-dependent regulation of ETB, suggest that this response may be mediated, at least in part, by other GCX components with mechanotransducing properties, such as HA or SAs [16]. It is also possible that a critical threshold of HS degradation is necessary to induce a statistically significant alteration in ETB expression.

Increased ETB protein expression coupled with decreased gene expression in response to 12 h of exposure to uniform SS implies that there may be a shift in overall ETB expression in HLMVECs occurring around 12 h of SS exposure. Consistent with the complex expression pattern of ET-1, exploring a broader range of SS intensities and exposure durations may help identify further modulatory effects on ETB expression. The duration required for translating mechanosensory stimuli into a biochemical response is poorly understood and likely variable across responses, and this could explain the observed differences between expressed protein and mRNA expression.

While GCX components such as HS are anchored to the cytoskeleton via transmembrane HSPGs [59], transmembrane protein ETB is not known to be directly connected to the cytoskeleton. However, ETB may interact with the cytoskeleton through a signaling pathway that modulates the release of NO through eNOS activation [30,36,60]. This indicates that SS dependence may rely on more than just HS-induced mechanotransduction. Krawczyk et al. found that the actin cytoskeleton was involved in transcriptional control of ETB via depolymerization of actin [61]. Binding of ET-1 to ETB in the endothelium begins a signaling cascade leading to actin–myosin interaction and SMC contraction [30]. Thi et al. have demonstrated the connection between the GCX and the actin cortical web just beneath the plasma membrane within the EC [59]. These findings suggest that, while alterations to the HS mechanotransduction pathway may still influence ETB regulation, additional pathways likely contribute, offering opportunities for further exploration beyond the scope of our current results.

Additionally, HS-dependent ETB expression may influence gene expression or post-translational regulation of ET-1. ETB is known to be concentrated in specific membrane microdomains such as caveolae and lipid rafts [62]. HS and glypican-1 have also been shown to cluster in lipid rafts when exposed to SS [63]. HS or HSPGs may cluster or organize ETB conformation or mobility.

### 4.4. Literature Comparison

These results show the SS dependence of ET-1 and may suggest that HS is partially involved in regulating ET-1. However, Morita et al. have demonstrated that SS-induced alterations in EC cytoskeleton structures are responsible for increased ET-1 synthesis in porcine aortic ECs [39] and the GCX is known to be connected to the cytoskeleton [59]. Our results from static culture treatment with Hep-III showed a small but statistically significant increase in ET-1 protein expression but not gene expression when HS is cleaved. Additionally, the SS-dependent increase in ET-1 gene expression seen under high SS conditions was reduced by Hep-III treatment. These results, coupled with the known cytoskeletal connection from Morita et al., suggest that reducing the mechanotransduction ability of HLMVECs may lead to a response in ET-1 expression, suggesting a potential HS dependence of ET-1 despite this result not being observed under all SS conditions.

ET-1 expression has been shown to be multi-phasic, with downregulation and upregulation both occurring in response to different SS magnitudes and varied SS exposure durations [64]. In contrast to our observations, Morawietz et al. and others have reported ET-1 mRNA expression as downregulated under long-term (8 and 24 h) exposure to 15 dynes/cm^2^ in human umbilical vein ECs (HUVECs) [25,65], while others, such as Morita et al., have reported upregulation of ET-1 in porcine aortic ECs under 5 dynes/cm^2^ for 6–12 h [39,52]. Other studies have also reported elevated ET-1 mRNA expression in porcine ECs under low SS conditions. Yoshizumi et al. and others have found that hemodynamic SS stimulates ET-1 secretion [26,52]. These observations align with our findings regarding post-translational regulation of ET-1. Notably, shear-dependent ET-1 expression in HLMVECs remains largely unexplored. Given the distinct gene regulation profiles between EC types and the differing native environments of HUVECs and HLMVECs, and the known variability in GCX structure across vessel types [66], it is plausible that HLMVECs exhibit a unique response to SS compared with HUVECs, which may lead to differential expression of ET-1 and ETB. Exploring their expression profiles in cell types across donors would add valuable insight into their function.

Additionally, application of SS in many cases was performed using a cone-and-plate viscometer that applies SS differently to the parallel-plate flow chamber used in this study. Cone-and-plate viscometers have been shown to introduce secondary flow characteristics, causing more disturbed flow conditions than parallel-plate flow chambers, which may lead to more varied or inconsistent cellular responses [20,67]. It may be the case that the method of SS application plays an important role in ET-1 gene regulation. The specific SS magnitude imparted on the cells also appears to be critical to the gene expression profile of ET-1.

The response of ETB to SS and its regulatory mechanisms are not yet well understood. ETB, a GPCR, belongs to a class of receptors known to be SS sensitive in ECs [29,30,68]. Morawietz et al. reported an increase in ETB mRNA in HUVECs following prolonged (24 h) SS exposure, which they attributed to an NO-dependent mechanism [25]. Similarly, Tang et al. observed elevated ETB mRNA levels in rat pulmonary microvascular endothelial cells after 12 h of SS exposure and noted an increase in ETB protein levels after 24 h [36]. Supporting these findings, Redmond et al. demonstrated upregulation of ETB mRNA under SS using a transcapillary culture model on bovine aortic ECs [37].

In addition to SS, ETB has also been shown to be modulated by other stimuli, including perfusion pressure, where increased pressure leads to upregulation of ETB mRNA [69,70], as well as cytokines, growth factors, including TNF-α and fibroblast growth factor, and low-density lipoproteins [71,72]. Our results confirm the SS dependence of ETB but yielded an opposite change in ETB mRNA than what Morawietz et al. and Tang et al. observed, likely due to the different SS exposure durations and cell type of our experiments. To the best of the authors knowledge, limited studies have explored ETB SS dependence in endothelial cells, with only a few groups, including Morawietz et al. and Tang et al., reporting relevant data.

The response of the GCX and its component HS on the EC surface to uniform SS is well understood. HS proteoglycan mRNA and its response to SS have not been as extensively studied. Gene expression of the HS proteoglycans perlecan and syndecan-1 was not significantly changed under exposure to 5, 15, and 25 dynes/cm^2^ of uniform SS, indicating that exposure to SS at these magnitudes for 12 h may not induce HS proteoglycan synthesis in ECs. Liu et al. demonstrated syndecan-1 upregulation due to short-term exposure to 4, 10, and 15 dynes/cm^2^ of SS in HUVECs with a peak in expression between 0.5 and 8 h, but this effect was reduced or eliminated by long-term exposure (24 h) [53]. They did not examine higher SS magnitudes or 12 h exposure to SS. Russo et al. demonstrated increased perlecan gene expression under 4 dynes/cm^2^ of SS for 4 h and a decrease in perlecan gene expression under 12 dynes/cm^2^ of SS for 4 h compared with static controls using rabbit aortic ECs [19]. These differences in SS magnitude and time as well as cell type may explain the differential results in our study. Time course studies would aid in clarifying the SS dependence of ETB, as the translation duration of hemodynamic force into biochemical action is not well understood. Investigating timepoints both shorter and longer than 12 h would improve our understanding of these results. Future studies should also investigate the role of glypican-1, a key HSPG that modulates nitric oxide production and endothelial remodeling [11,12]. While the role of glypican-1 in mechanotransduction and vascular homeostasis makes it a logical candidate for investigation, resource and time constraints prevented its inclusion in the current study.

In conclusion, further research is needed to explore ET-1 secretion dynamics, the effects of ETB antagonism, and additional mechanisms of GCX disruption. This study did not assess ET-1 secretion through ELISA or examine intracellular protein localization, both of which are important for clarifying the disconnect between gene upregulation and protein-level changes observed in our data. Future studies employing HS-specific knockdown models, such as perlecan or syndecan-1 CRISPR, may help identify the key HSPGs involved in ETB regulation. Moreover, analyzing HS sulfation patterns, e.g., through 3-O sulfation assays, could reveal specific modifications that influence ETB localization and function. Investigating ETB internalization and recycling pathways under SS will also provide insights into HS-mediated receptor trafficking. Additionally, atomic force microscopy (AFM) could be employed to quantify the binding forces between ET-1 and ETB in both healthy and diseased GCX states, offering a unique perspective on whether the GCX directly modulates ETB production or release. Together, these approaches will further elucidate the complex interplay between the GCX and ETB under SS conditions.

## 5. Conclusions

In summary, the complex relationship between ET-1, ETB, and HS and its proteoglycans was investigated. We found clear SS dependence of both ET-1 and ETB and that a reduction in ECs’ mechanotransduction ability by HS degradation is potentially involved in regulation of both ET-1 and ETB in specific cases, though to differing degrees and without conclusive results. SS triggers HS-independent transcriptional upregulation of ET-1, but not an increase in protein levels within the cell and on the cell surface, suggesting post-translational or secretion-based regulation of ET-1. We showed that SS induces gene downregulation of ETB and protein upregulation, demonstrating that ETB is SS dependent and responsive to SS at both transcriptional and protein levels. Little research has been carried out in the area of ETB SS dependence, and this research adds valuable insight into the regulation of ETB. SS is vital for vascular regulation and GCX health and homeostasis [3]. Degradation of HS may reduce the SS dependence of ETB at low and physiological SS magnitudes by disrupting the mechanotransduction ability of the endothelium. Taken together, these results indicate that HS may facilitate ETB membrane localization, stability, or trafficking, though this response was minimal. While not strongly correlated, some change in ETB expression between enzyme-treated and untreated samples was seen, indicating the hypothesized cooperation with HS and likely with other GCX components. Finally, we demonstrated a SS-dependent increase in HS expression through post-translational remodeling. These observations suggest a complex relationship between SS, GCX integrity, ETB function, and ET-1 regulation within the cell. Understanding these complex relationships and further investigating the SS dependence of ETB may aid in the diagnosis and treatment of vascular diseases involving regulation of ETB and changes in SS such as hypertension.

## Figures and Tables

**Table 1:** Summary of observed results for HLMVECs exposed to SS and 15 mU/mL Hep-III. Arrows indicate an increase (↑) or decrease (↓) in protein or gene expression.

Parameter	5 Dynes/cm^2^	15 Dynes/cm^2^	25 Dynes/cm^2^
ET-1 Protein (ICC)	No change	No change	No change
*EDN1* Gene (qPCR)	↑ HS independent	↑ HS independent	↑ Partial HS dependence
	↑ SS dependent	↑ SS dependent	↑ SS dependent
ETB Protein (ICC)	↑ Partial HS dependence	↑ Partial HS dependence	↑ HS independent
	↑ SS dependent	↑ SS dependent	↑ SS dependent
*EDNRB* Gene (qPCR)	No change	↓ HS independent	↓ HS independent
		↓ SS dependent	↓ SS dependent
Surface HS (ICC)	↑ SS induced	↑ SS induced	↑ SS induced
HS Proteoglycan Gene (qPCR)	No change	No change	No change

**Figure A1:**
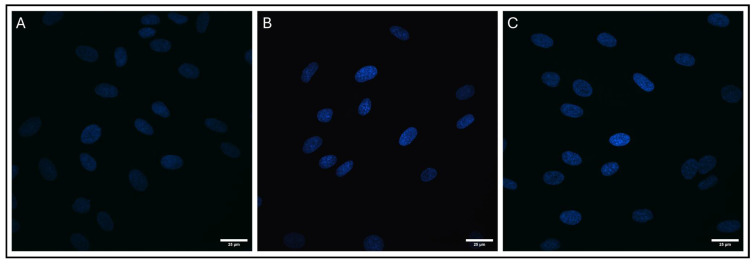
Representative negative control images (secondary antibody only) for (**A**) ET-1, (**B**) ETB, and (**C**) HS.

## Data Availability

The original raw image and qPCR data and all analysis tools used in this study, including ImageJ FIJI macros and CellProfiler pipelines, are openly available in FigShare at DOI: 10.6084/m9.figshare.29508200.

## Abbreviations

The following abbreviations are used in this manuscript:

GCX	Endothelial Glycocalyx
HLMVEC	Human Lung Microvascular Endothelial Cells
ECs	Endothelial Cells
GAGs	Glycosaminoglycans
SS	Shear Stress
UF	Uniform Flow
HS	Heparan Sulfate
ET-1	Endothelin-1
ETB	Endothelin B Receptor
HSPG	Heparan Sulfate Proteoglycan

## Appendix A

### Appendix A.1

**Table A1:** Primer sequences.

Primer Target	Forward Sequence	Reverse Sequence
*EDN1*	TCAACACTCCCGAGCACGTT	TCACGGTCTGTTGCCTTTGT
*EDNRB*	ATGATCACCTAAAGCAGAGACGG	CAGAGGGCAAAGACAAGGAC
*HSPG2*	AGCATCTCAGGAGACGACCT	GAAATTCACCAGGGCTCGGA
*SDC1* [47]	CTGCCGCAAATTGTGGCTAC	TGAGCCGGAGAAGTTGTCAGA
*GAPDH*	ACCATCTTCCAGGAGCGAGA	GACTCCACGACGTACTCAGC
